# Transcriptome profiling of *Pinus radiata *juvenile wood with contrasting stiffness identifies putative candidate genes involved in microfibril orientation and cell wall mechanics

**DOI:** 10.1186/1471-2164-12-480

**Published:** 2011-10-01

**Authors:** Xinguo Li, Harry X Wu, Simon G Southerton

**Affiliations:** 1 CSIRO Plant Industry, GPO Box 1600, Canberra ACT 2601, Australia; 2 Umeå Plant Science Centre, Dept. Forest Genetics and Plant Physiology, Swedish University of Agricultural Sciences, SE-901 83 Umeå, Sweden

## Abstract

**Background:**

The mechanical properties of wood are largely determined by the orientation of cellulose microfibrils in secondary cell walls. Several genes and their allelic variants have previously been found to affect microfibril angle (MFA) and wood stiffness; however, the molecular mechanisms controlling microfibril orientation and mechanical strength are largely uncharacterised. In the present study, cDNA microarrays were used to compare gene expression in developing xylem with contrasting stiffness and MFA in juvenile *Pinus radiata *trees in order to gain further insights into the molecular mechanisms underlying microfibril orientation and cell wall mechanics.

**Results:**

Juvenile radiata pine trees with higher stiffness (HS) had lower MFA in the earlywood and latewood of each ring compared to low stiffness (LS) trees. Approximately 3.4 to 14.5% out of 3, 320 xylem unigenes on cDNA microarrays were differentially regulated in juvenile wood with contrasting stiffness and MFA. Greater variation in MFA and stiffness was observed in earlywood compared to latewood, suggesting earlywood contributes most to differences in stiffness; however, 3-4 times more genes were differentially regulated in latewood than in earlywood. A total of 108 xylem unigenes were differentially regulated in juvenile wood with HS and LS in at least two seasons, including 43 unigenes with unknown functions. Many genes involved in cytoskeleton development and secondary wall formation (cellulose and lignin biosynthesis) were preferentially transcribed in wood with HS and low MFA. In contrast, several genes involved in cell division and primary wall synthesis were more abundantly transcribed in LS wood with high MFA.

**Conclusions:**

Microarray expression profiles in *Pinus radiata *juvenile wood with contrasting stiffness has shed more light on the transcriptional control of microfibril orientation and the mechanical properties of wood. The identified candidate genes provide an invaluable resource for further gene function and association genetics studies aimed at deepening our understanding of cell wall biomechanics with a view to improving the mechanical properties of wood.

## Background

Wood cell (such as tracheids and fibres) initials are produced by the vascular cambium and subsequently undergo cell expansion, primary cell wall biosynthesis, secondary wall deposition, lignification, and finally programmed cell death [1,2]. In perennial woody plants, secondary xylem (wood) is derived from the annual activity of the vascular cambium, with wood laid down in different seasons and tree ages frequently having distinct mechanical properties [3-5], particularly in gymnosperms [5]. The mechanical properties of secondary xylem not only provide support for woody plants to maintain their shape, resist maturation stress (gravity), and respond to various environmental forces (wind, snow, etc); they also affect the suitability of wood for different commercial applications. A number of factors influence the mechanical properties of wood, including individual cell walls, anatomical structure, cell-cell adhesion and cell turgidity [6]. The mechanical properties of plant cell walls also play a crucial role in cell expansion [7,8], tissue or organ morphogenesis [9] and responses to various signals [10,11]. Understanding wood biomechanics at the cell and organ levels will provide unique insights into many biological processes in plants, such as cell wall biosynthesis and wood formation.

The mechanical properties of plant cell walls and organs are largely controlled by the architecture of the cytoskeleton [6,10]. Of the three main types of cytoskeleton polymers, microtubules are the stiffest while actin filaments are the least rigid [10]. Tethering of galactose residues in xyloglucans to cellulose microfibrils is essential for mechanical strength such as tensile properties of primary cell walls [6,12] and expansion relies on cooperation between specific expansins and XETs [13]. Previous studies have showed that cellulose microfibril orientation dominates mechanical properties in both primary and secondary cell walls of xylem [14-17], particularly microfibril angle (MFA) in the middle layer (S2) of secondary cell walls. In *Eucalyptus*, MFA alone has been estimated to account for 86% of the variation in wood stiffness with an additional 10% to be influenced by wood density [16].

The complex nature of the genetic control of wood properties was first showed by quantitative trait loci (QTL) mapping. Several QTLs have been identified that contribute to MFA and wood stiffness in pines [18-20], eucalypts [21-23] and poplar [24]. Recently several individual genes have been found to play roles in the regulation of microfibril orientation and mechanical properties of xylem cell walls. A *β-tubulin *gene has been found to influence cellulose microfibril orientation in wood cell walls of *Eucalyptus *[25]. Two fasciclin-like arabinogalactan proteins (FLA11 and FLA12) have been found to affect MFA and tensile stiffness in eucalypts and Arabidopsis by altering cellulose deposition and the integrity of the cell wall matrix [26]. Association studies have recently showed single nucleotide polymorphisms (SNPs) in a number of cell wall-related genes that influence MFA in loblolly pine (*α-tubulin, COMT2, CCR1*) [27], radiata pine (*RAC13, SuSy*) [28], white spruce (*glycosyl hydrolase 10*) [29] and *Eucalyptus nitens *(*CCR*) [30]. Furthermore, associations between allelic variation and other mechanical properties of wood have also been observed, including stiffness (*COMT*) and density (*PAL1 *and *PCBER2*) in radiata pine [28] and specific gravity (*CAD *and *SAMS2*) in loblolly pine [27]. While the research described above provides important insights into the molecular mechanisms of microfibril orientation and mechanical properties of wood cell walls, genes involved in the regulation of cell wall mechanics remain poorly characterised at the transcriptome level.

Radiata pine (*Pinus radiata *D.Don) is the most important conifer species in commercial forest plantations in Australia and several other countries and is grown primarily for structural timber. To gain further insight into the molecular control of microfibril orientation and cell wall mechanics, cDNA microarrays were used to investigate differential gene expression in radiata pine developing xylem with contrasting stiffness and MFA. Radiata pine trees with contrasting wood stiffness were selected in two progeny trials using acoustic velocity with an IML electronic hammer [31]. Stiffness, MFA and other wood property traits were further assessed in wood cores of the sampled trees using SilviScan 2 technology [32]. This high resolution technology can measure the properties of wood produced in different seasons and different years, and allowed us to compare gene expression and wood properties (such as MFA and stiffness) in a single season. The aim of this study was to identify putative candidate genes involved in the regulation of microfibril orientation and cell wall mechanics in wood cells.

## Results

### Selection of sampled trees with contrasting wood stiffness

**Figure 1:**
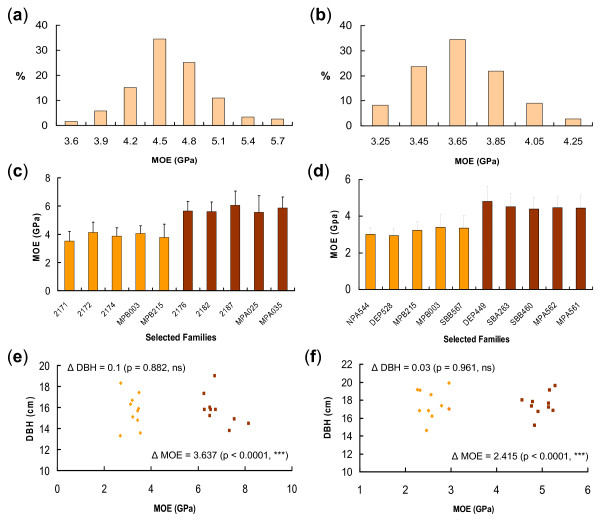
**Measurement of wood stiffness in standing trees using acoustic velocity**. Wood stiffness or the longitudinal modulus of elasticity (MOE) of standing trees was measured by acoustic velocity using an IML hammer. Average stiffness of trees in each family showed a roughly normal distribution in the Flynn (a) and Kromelite (b) trials. Ten families with the highest (HS) and lowest stiffness (LS) were selected and their stiffness variations are shown for the Flynn (c) and Kromelite (d) sites. Two individuals were further sampled in each selected family to maximize the stiffness variation within the Flynn (e) and Kromelite (f) trials. Average wood stiffness of the 10 sampled trees with HS was significantly higher than that of the 10 LS trees (P-values ≤ 0.0001) in both trials, but average diameter at breast height (DBH) was very similar.

Measurements of acoustic velocity of standing trees in the two progeny trials showed that variation in juvenile wood stiffness, or the longitudinal modulus of elasticity (MOE) [33], had roughly normal distributions at the family level (Figure 1a, b). Continuous variation of wood stiffness in the two trials is a typical feature of quantitative traits. Overall average MOE in the Flynn and Kromelite trials were 4.66 and 3.72 GPa with standard deviations (SD) of 0.42 and 0.22, respectively. The lower MOE in the Kromelite trial may be due to the higher tree growth rate in that trial, as there is a negative correlation between MOE and tree growth in radiata pine [34,35].

In each trial five families with the highest MOE and five families with the lowest MOE were selected for further study (Figure 1c, d), thus between 4.0 and 9.1% of the total families were selected from each trial. The two groups of selected families had large differences in wood stiffness (Figure 1c, d). The families MPB215 and MPB003 were among the five low stiffness (LS) families selected in both trials, which is likely to be due to the moderate to high heritability of juvenile wood stiffness [35-39]. Two individuals with higher (or lower) MOE and above average growth rates were sampled from each selected family for further study (Figure 1e, f). Developing xylem tissues of these individuals were collected at three different seasons (spring, summer and autumn), and total RNAs were extracted for microarray experiments.

### SilviScan profiling of microfibril angle and other wood traits

**Figure 2:**
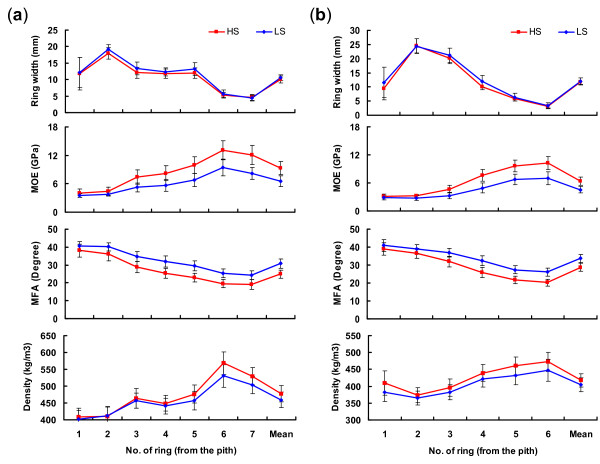
**Variation in mechanical and other wood properties between the two groups of sampled trees: individual rings**. Ring width, modulus of elasticity (MOE), microfibril angle (MFA) and wood density were measured at a high-resolution in wood cores from the 20 sampled trees using SilviScan 2. Wood traits in each ring were compared between wood with contrasting stiffness and MFA, including ring width, MOE, MFA and density in Flynn (a) and Kromelite (b). Error bars represent the standard deviation of the mean value of each trait. Variation in both MOE and MFA between the two groups of sampled trees is statistically significant while no significant variation appears in ring width and density (P-values ≤ 0.05).

The acoustic velocity-based method using an IML hammer provides an overall measurement of wood stiffness (or MOE) of a standing tree. Wood cores of the 20 high stiffness (HS) and low stiffness (LS) trees selected in each trial were further analysed using SilviScan 2. This high resolution technology measured MFA, MOE and wood density along wood cores in earlywood and latewood of each ring formed in different seasons of each year. The three wood traits were compared in each ring between the HS and LS trees (Figure 2). Annual growth rates as seen in the width of each ring were similar between the two groups (P-values ≤ 0.05), suggesting growth rate of the sampled trees should have a limited impact on comparisons of wood stiffness. In the first 3-4 rings (from the pith) the differences in MFA, MOE and wood density between the two groups were not statistically significant; while from ring 4 or 5 onwards MFA in each ring of HS trees was significantly lower and MOE significantly higher than that in LS trees (P-values ≤ 0.05) (Figure 2). In contrast, wood density in these rings was not significantly different between the two groups. Thus, the 20 standing trees with contrasting stiffness based on acoustics were further confirmed by the SilviScan measurements. The SilviScan results also showed that the two groups of trees had contrasting stiffness and MFA in individual rings produced after 3 or 4 years. Thus, when developing xylem tissues were sampled for microarray experiments from the two plantations at year 6 and 7, respectively, the single ring produced in the two groups of sampled trees had contrasting stiffness and MFA. These provided a basis for the relatively precise analysis of wood traits (wood stiffness and MFA) and gene expression in the same ring. Thus, differential gene expression between the two groups of sampled trees is expected to be largely related to their contrasting stiffness and MFA rather than wood density.

**Figure 3:**
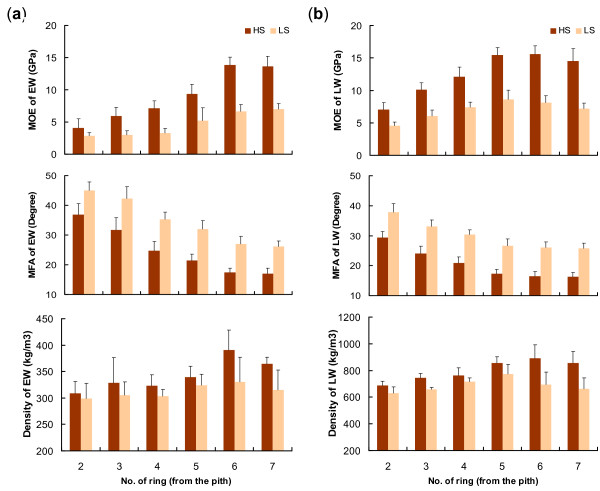
**Variation in mechanical and other wood properties between the two groups of sampled trees in the Flynn trial: earlywood and latewood in each ring**. Microfibril angle (MFA), modulus of Elasticity (MOE) and wood density in wood cores from the 20 sampled trees were measured by SilviScan 2. Wood variation between the two groups of sampled trees was compared in earlywood (EW) (a) and latewood (LW) (b) of each ring for MOE, MFA and density. Error bars represent the standard deviation of the mean value of each trait. Variation in MFA and MOE is statistically significant (P-values ≤ 0.05) but it is not statistically significant for wood density variation in all rings.

For comparisons between gene expression and wood formed in a particular growing season, SilviScan data was analysed in different seasons of each year. Three wood properties (MFA, MOE and density) were compared between HS and LS trees in both trials in earlywood (EW) and latewood (LW) (Figure 3 and Additional file 1). MFA showed a marked decline while MOE increased with tree age (up to 6 yrs) in the EW and LW of both HS and LS trees. Compared to the 10 trees with LS (IML-based) in both trials, the 10 HS trees (IML-based) had a significantly higher MOE (SilviScan) and lower MFA in the EW and LW of each ring (P-values ≤ 0.05). In the two groups of sampled trees, LW stiffness of each ring was generally greater than that of EW stiffness; while MFA of LW in each ring was consistently lower than that of EW in both trials (Figure 3 and Additional file 1). Unlike MOE and MFA, wood density was not significantly different between the two groups of trees in both EW and LW, except for a few rings produced in the earlier years in the Kromelite trial (Additional file 1). In summary, the two groups of sampled trees produced annual rings and both EW and LW with contrasting MOE and MFA, but similar wood density (particularly in rings produced in the later years). These results further suggested that differences in xylem gene expression in different seasons between the two groups of sampled trees would most likely be related to wood stiffness and MFA rather than density.

**Figure 4:**
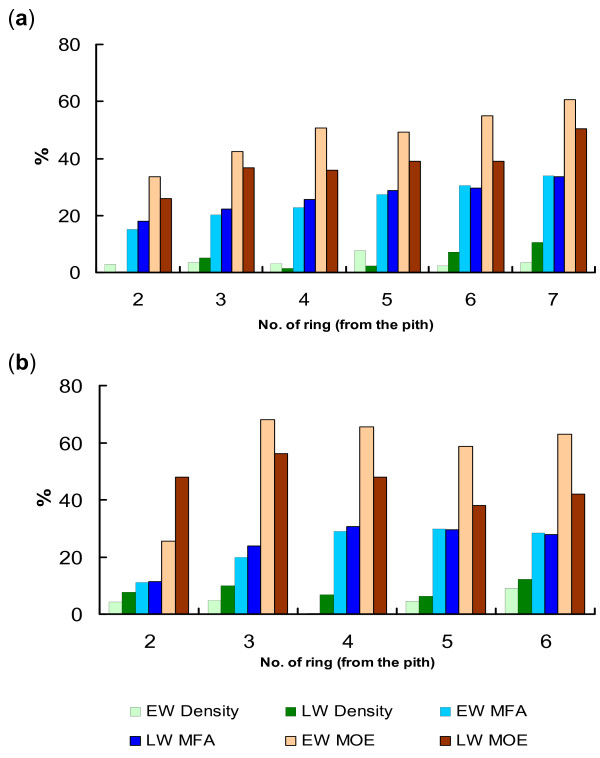
**Relative variation of mechanical and other wood properties between 10 selected families with contrasting stiffness: earlywood and latewood in each ring**. Modulus of elasticity (MOE), microfibril angle (MFA) and density were measured by SilviScan 2 in 40 (Flynn) or 60 (Kromelite) wood cores collected from 10 families with the highest (HS) and lowest stiffness (LS) based on acoustics. Relative variation between the two groups of selected families in the Flynn (a) and Kromelite trials (b) is presented for earlywood (EW) and latewood (LW) using (HS-LS)/LS×100% (for MOE and density) or (LS-HS)/HS×100% (for MFA).

In order to obtain more reliable patterns of wood property variation between HS and LS trees, an additional 20 and 40 trees from the 10 HS and LS families in the Flynn and Kromelite trials, respectively, were measured by SilviScan and the data combined with SilviScan data from the 20 trees used for the microarray experiments. The magnitude of MFA variation between the HS and LS trees in both trials was similar in EW and LW of each ring, particularly in the outer rings closest to the sampling time (Figure 4). In contrast, in both trials variation in MOE between these trees was typically higher in EW than in LW in each ring, suggesting that EW tissues formed in spring could be a major contributor to overall variation in wood stiffness. However, wood density had an opposite pattern compared to wood stiffness. In the Kromelite trial, the density difference between the HS and LS trees tended to be higher in LW than in EW of each ring; while in the Flynn trial greater density differences were also observed in LW in the two rings closest to the bark.

### Differential gene transcription in wood with distinct stiffness and MFA

**Figure 5:**
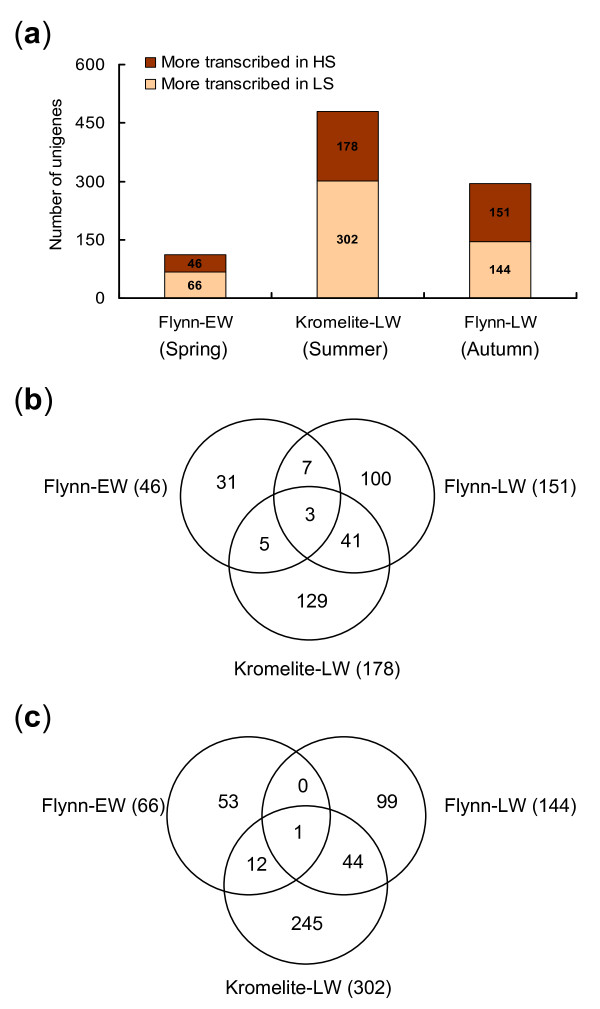
**Differentially transcribed unigenes identified in the Flynn and Kromelite trials**. Unigenes differentially transcribed in wood with contrasting stiffness and microfibril angle were identified using cDNA microarrays containing 3, 320 xylem unigenes. The number of unigenes identified at different sampling seasons in the two trials (a), unigenes preferentially transcribed in the highest stiffness (lower microfibril angle) (b) and lowest stiffness wood (higher microfibril angle) (c) were compared. Identified unigenes common to developing xylem tissues collected in different seasons are also indicated in the figures.

Transcripts differentially accumulated in radiata pine developing xylem with contrasting wood stiffness and MFA were identified in the Flynn trial in spring (Flynn-EW) and autumn (Flynn-LW) and the Kromelite trial in summer (Kromelite-LW). Of 3, 320 xylem unigenes present on the cDNA microarrays, 3.4% were differentially regulated in Flynn-EW, including 46 and 66 unigenes preferentially transcribed in HS (low MFA) and LS (high MFA), respectively (Figure 5a). In contrast, more unigenes (8.9%, 2.6 times) were differentially transcribed in the same trees in autumn (LW), including 151 unigenes preferentially transcribed in HS and 144 in LS trees (Figure 5a). However, the largest proportion of differentially accumulated transcripts was found in trees (14.5%) sampled in summer (LW) in the Kromelite trial. Thus, LW tissues had 3-4 times more differentially accumulated transcripts than EW tissues, suggesting that expression of genes regulating wood stiffness and microfibril orientation is impacted by the season.

While the number of differentially transcribed unigenes was 3-4 times higher in LW than in EW (Figure 5a), the greatest differences in wood stiffness (MOE) in rings formed in the sampling years were observed in EW laid down in spring (Figure 4). This suggests that the number of differentially transcribed unigenes alone does not explain the seasonal pattern of MOE variation. Comparisons of differentially accumulated unigenes in the Flynn trial showed that only 10 (or one) are common to both EW (spring) and LW (autumn) in juvenile wood with HS and low MFA (or LS and high MFA) (Figure 5b, c). Similarly, only eight (or 13) unigenes were common in both EW (spring) in Flynn and LW (summer) in Kromelite. In contrast, considerably more differentially regulated unigenes were conserved in LW tissues sampled in autumn (Flynn) and summer (Kromelite), including 43 unigenes for HS (low MFA) and 45 for LS (high MFA) wood (Figure 5b, c). Taken together, these results suggested that distinct sets of genes control microfibril orientation and cell wall mechanics in different seasons.

**Figure 6:**
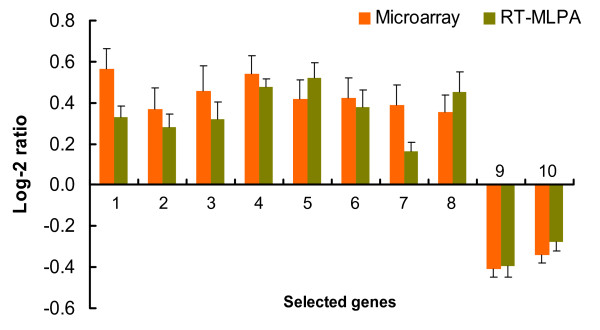
**Validation of microarray expression of selected genes by RT-MLPA**. A total of 10 differentially regulated genes were selected in the validation using reverse transcriptase-multiplex ligation dependent probe amplification (RT-MLPA). These genes included eight genes preferentially transcribed in wood with high stiffness (HS) and lower microfibril angle (LM): 1- *cellulose synthase 3 *(*PrCesA3*); 2- *chitinase-like*; 3- *protective protein for beta-galactosidase *(*PPBG*); 4- *cinnamate 4-hydroxylase *(*C4H*); 5- *phytochrome*; 6- *PrCesA11*; 7- *CCoAOMT*; 8- *transmembrane serine protease 9 *(*TSP9*), and two genes selected in wood with low stiffness (LS) and higher microfibril angle (HM), 9- *FadR *and 10- *T12C24.1*. Log-2 ratios (HS/LS) of gene expression were calculated, and the values > 0 and < 0 indicate genes preferentially accumulated in HS (LM) and LS (HM) wood, respectively. Error bars represent the standard deviation of the mean log-2 ratio.

Microarray expression of 10 differentially transcribed unigenes (eight from HS and low MFA, and two from LS and high MFA) selected in the Flynn-LW experiment were validated by RT-MLPA using the same RNA samples as for the microarray experiments. Transcript accumulation measured by the RT-MLPA method was relatively consistent with the microarray results for all 10 validated genes (Figure 6), particularly in the ranking of expression magnitude, suggesting the microarray experiments in this study were sufficiently reliable for the identification of candidate genes that may influence juvenile wood stiffness and MFA.

### Functional analysis of differentially accumulated transcripts

A total of 307 and 446 xylem unigenes (after redundant unigenes were removed) preferentially transcribed in juvenile wood with HS (low MFA) and LS (high MFA), respectively, were identified in the three microarray experiments. More than 64% of the unigenes had homologs in the UniProt known protein [40] and RefSeq [41] databases (tblastx, E-value < 1e-5) (Table 1). Functional analysis showed that 52-59% of the unigenes had been assigned a gene ontology (GO) term, while the remaining 41-48% were functional unknowns (Table 1). Among the differentially transcribed unigenes with assigned GO terms, the majority were classified as molecular functions (86-93%) or biological processes (81-82%), with fewer as cellular components (61-64%) (Table 1). Slightly more GO terms were assigned to unigenes preferentially transcribed in wood with HS and low MFA (59.3%) compared to LS trees with high MFA (52%), and more genes with molecular functions and/or involved in cellular components are preferentially transcribed in LS wood with high MFA (Table 1).

**Table 1:** Functional annotation of unigenes preferentially transcribed in juvenile wood with contrasting stiffness and microfibril angle *^a ^*

	*Unigenes*	*Blast matches^b ^*		*GO terms assigned* ^c^			
			
		UniProt	UniProt&RefSeq	Total	CC	MF	BP
HS (LM)	307	196 (63.8%)	200 (65.1%)	182 (59.3%)	111 (61.0%)	156 (85.7%)	149 (81.9%)
LS (HM)	446	284 (63.7%)	286 (64.1%)	232 (52.0%)	149 (64.1%)	215 (92.7%)	188 (81.0%)

^a^Juvenile wood included two types: wood with high stiffness (HS) and low microfibril angle (LM), and wood with low stiffness (LS) and high microfibril angle (HM). ^b^Refer to the matches in the UniProt known protein and RefSeq databases (tblastx, E-value < 1e-5). ^c^CC- cellular component; MF- molecular function; BP- biological process. Percentage (%) of CC, MF and BP were calculated using the total unigenes assigned with GO terms.

Further comparisons of lower level GO terms assigned to the differentially transcribed unigenes showed distinct differences between juvenile wood with contrasting stiffness and MFA in 17 sub-categories (Table 2). In terms of cellular components, genes involved in vacuolar membrane and proton-transporting two sector ATPase complexes had higher representation in HS wood with low MFA; whereas genes with functions in external encapsulating structure were preferentially transcribed in LS wood with high MFA. Genes more abundantly transcribed in trees with HS and low MFA included more genes with different molecular functions, such as binding activities (actin, copper ion, heme, tetrapyrrole and transition metal ion), translation elongation factors and transmembrane transporters. In contrast, only two sub-categories of molecular functions (coenzyme binding and carbon-oxygen lyase activity) had higher representation in juvenile wood with LS and high MFA. In the HS wood (low MFA), genes involved in translational elongation, transport (ion, electron and cation), stress responses and ribonucleotide biosynthetic process were more highly transcribed; whereas LS wood with high MFA had increased transcription of genes involved in different metabolic and developmental processes and RNA processing.

**Table 2:** Comparisons of gene ontology (GO) terms of unigenes differentially transcribed in juvenile wood with contrasting stiffness and microfibril angle

***GO No***.	*GO Terms*	*Class^a^*	*HS ^b^*	*LS ^b^*	*P-value*
GO:0030312	External encapsulating structure	CC	0	15	0.028
GO:0016469	Proton-transporting two-sector ATPase complex	CC	9	0	0.037
GO:0005774	Vacuolar membrane	CC	16	2	0.034
GO:0003779	Actin binding	MF	9	0	0.037
GO:0022804	Active transmembrane transporter activity	MF	32	11	0.041
GO:0016835	Carbon-oxygen lyase activity	MF	0	13	0.042
GO:0050662	Coenzyme binding	MF	0	20	0.013
GO:0005507	Copper ion binding	MF	9	0	0.037
GO:0020037	Heme binding	MF	16	2	0.034
GO:0004497	Monooxygenase activity	MF	9	0	0.037
GO:0004673	Protein histidine kinase activity	MF	9	0	0.037
GO:0046906	Tetrapyrrole binding	MF	16	2	0.034
GO:0046914	Transition metal ion binding	MF	68	35	0.048
GO:0003746	Translation elongation factor activity	MF	13	0	0.016
GO:0000155	Two-component sensor activity	MF	9	0	0.037
GO:0019752	Carboxylic acid metabolic process	BP	19	47	0.050
GO:0006812	Cation transport	BP	29	8	0.037
GO:0042180	Cellular ketone metabolic process	BP	19	47	0.050
GO:0044255	Cellular lipid metabolic process	BP	3	33	0.005
GO:0032502	Developmental process	BP	13	42	0.023
GO:0022900	Electron transport chain	BP	16	2	0.034
GO:0006631	Fatty acid metabolic process	BP	0	17	0.019
GO:0006811	Ion transport	BP	29	8	0.037
GO:0032787	Monocarboxylic acid metabolic process	BP	0	26	0.004
GO:0034470	ncRNA processing	BP	0	15	0.028
GO:0006082	Organic acid metabolic process	BP	19	47	0.050
GO:0009651	Response to salt stress	BP	19	4	0.049
GO:0050896	Response to stimulus	BP	91	51	0.040
GO:0006950	Response to stress	BP	68	33	0.032
GO:0009260	Ribonucleotide biosynthetic process	BP	19	4	0.049
GO:0006396	RNA processing	BP	3	26	0.015
GO:0006414	Translational elongation	BP	13	0	0.016

^a^CC: cellular component; MF: molecular function; BP: biological process. ^b^HS: wood with high stiffness and low microfibril angle; LS: wood with low stiffness and high microfibril angle; The number of GO terms assigned in the identified unigenes was normalized at 1, 000 unigenes.

### Putative candidate genes for microfibril orientation and wood stiffness

To identify putative candidate genes involved in the regulation of wood stiffness and microfibril orientation, differentially accumulated transcripts identified in a single microarray experiment were examined in the other two microarray experiments. In the two experiments with LW tissues, 46.5% of transcripts identified in Flynn-LW (autumn) were confirmed in Kromelite-LW (summer), and 30.7% transcripts identified in Kromelite-LW (summer) were detected in Flynn-LW (autumn). In contrast, fewer genes identified in Flynn-EW (spring) were confirmed in Flynn-LW (19.4%) and Kromelite-LW (28.7%). This is likely to be caused by differential gene expression in wood tissues formed in different seasons.

A total of 51 and 57 candidate genes were identified with differential transcription in juvenile wood with HS (low MFA) and LS (high MFA), respectively, in at least two of the three microarray experiments (Additional file 2). Among these candidate genes 43 were functional unknowns based on the UniProt known protein and RefSeq databases. The remaining candidate genes for HS (and low MFA) with relatively clear functions are listed in Table 3 (29 genes for HS - low MFA) and Table 4 (34 for LS - high MFA). Of the 29 candidate genes for HS (low MFA) eight genes are involved in secondary cell wall formation, including four lignin genes (*C4H, C3H, CAD *and *PCBER*) and four secondary wall *cellulose synthases *(*PrCesA1, 3, 7, 11*) [42]. Other genes involved in cell wall synthesis included *sucrose synthase *(*SuSy*) and genes involved in actin filament (*actin depolymerising factor *and *actin*) and microtubule development (*tubulin beta-3*). In contrast, only three of the 34 candidate genes preferentially transcribed in LS (high MFA) wood had a clear function in cell wall development (*cellulase, pectate lyase, peroxidase*) and none were specifically involved in secondary cell wall synthesis.

**Table 3:** Putative candidate genes with known functions preferentially transcribed in juvenile wood with high stiffness and low microfibril angle

*Candidate genes *	***GenBank Accession***^a^	***Flynn-EW ***^b^	***Flynn-LW ***^b^	***Kromelite- LW ***^b^	*Mean ratio*	*Mean P-value*
*Actin depolymerizing factor *	FE521693	1.74 (0.048)	1.82 (0.051)	1.93 (0.042)	1.83	0.047
*C4H *	FE519540	1.64 (0.047)	1.71 (0.021)	1.40 (0.014)	1.59	0.027
*Elicitor inducible beta-1, 3-glucanase*	FE518343	2.18 (0.050)	1.71 (0.049)	1.38 (0.042)	1.76	0.047
*Phytochrome *	FE524197	1.70 (0.010)	1.58 (0.027)	1.40 (0.037)	1.56	0.025
*Tubulin beta-3*	FE519523	1.74 (0.048)	1.58 (0.050)	1.62 (0.014)	1.65	0.037
*Actin*	FE518593		1.52 (0.037)	1.32 (0.012)	1.42	0.025
*ADP ATP carrier protein *	FE519476		1.76 (0.048)	1.47 (0.035)	1.61	0.042
*Alpha/beta hydrolase *	FE524486		1.32 (0.043)	1.34 (0.053)	1.33	0.048
*C3H *	FE520787		1.41 (0.044)	1.51 (0.036)	1.46	0.040
*CAD *	FE520918		1.49 (0.048)	1.34 (0.051)	1.42	0.050
*Chaperone GrpE type 1*	FE523866	1.46 (0.049)		1.46 (0.051)	1.45	0.050
*Chloroplast DnaJ-like 2*	FE522001		1.48 (0.046)	1.61 (0.050)	1.54	0.048
*Cytochrome b5*	FE519722		1.39 (0.047)	1.41 (0.039)	1.40	0.043
*Cytokinin-binding protein*	FE518619		1.41 (0.047)	1.57 (0.041)	1.49	0.044
*D-cysteine desulfhydrase *	FE523039		1.34 (0.042)	1.31 (0.052)	1.32	0.047
*Defender against apoptotic death 1*	FE523621		1.41 (0.039)	1.36 (0.053)	1.39	0.046
*eIF-2-gamma*	FE521528		1.41 (0.038)	1.40 (0.050)	1.41	0.044
*Hin1 containing protein*	FE518865		1.45 (0.045)	1.55 (0.028)	1.50	0.037
*Light-inducible protein*	FE523148		1.51 (0.040)	1.65 (0.050)	1.58	0.045
*PCBER*	FE523120		1.33 (0.036)	1.32 (0.027)	1.33	0.032
*PrCesA1*	FE521029		1.51 (0.054)	1.46 (0.012)	1.49	0.033
*PrCesA11*	FE524308		1.76 (0.051)	1.47 (0.047)	1.61	0.049
*PrCesA3*	FE520377		1.44 (0.049)	1.49 (0.046)	1.47	0.048
*PrCesA7*	FE518578	2.41 (0.045)	1.40 (0.033)		1.91	0.044
*SDL-1 protein*	FE523204	1.44 (0.050)	1.34 (0.002)		1.39	0.025
*Sucrose synthase*	FE519487		1.38 (0.054)	1.31 (0.045)	1.35	0.050
*Vacuolar ATPase subunit *	FE520935		1.39 (0.016)	1.47 (0.051)	1.43	0.034
*Vignain precursor *	FE522061		1.42 (0.008)	1.88 (0.037)	1.65	0.023
*Zinc finger, C2H2-type*	FE522289		1.31 (0.038)	1.49 (0.039)	1.40	0.039

**^a^**GenBank accession number for the representing EST of each gene.**^b^**Three microarray experiments: Flynn-EW (earlywood in Flynn, spring), Flynn-LW (latewood in Flynn, autumn) and Kromelite-LW (latewood in Kromelite, summer). Gene expression ratios with (P-values) are presented.

**Table 4:** Putative candidate genes with known functions preferentially transcribed in juvenile wood with low stiffness and high microfibril angle

*Candidate genes*	***GenBank Accession***^a^	***Flynn-EW ***^b^	***Flynn-LW ***^b^	***Kromelite- LW ***^b^	*Mean ratio*	*Mean P-value*
*10 kDa chaperonin protein *	GO269358	1.50 (0.054)		1.33 (0.045)	1.42	0.050
*ALG2-interacting protein*	FE519827		1.42 (0.021)	1.33 (0.049)	1.38	0.035
*Annexin 1c*	FE519074		1.40 (0.053)	1.37 (0.048)	1.39	0.051
*Brix domain*	FE522600	1.46 (0.051)		1.39 (0.044)	1.43	0.048
*C13 endopeptidase NP1 *	FE520715		1.37 (0.038)	1.37 (0.045)	1.37	0.042
*CC-NBS-LRR resistance-like *	FE520054		1.33 (0.051)	1.35 (0.048)	1.34	0.050
*Cellulase*	FE520483		1.36 (0.004)	1.34 (0.054)	1.35	0.029
*Cyclin-like F-box*	FE520391		1.45 (0.021)	1.28 (0.052)	1.37	0.037
*Disease resistance gene*	FE523993	1.22 (0.052)		1.25 (0.046)	1.24	0.049
*DnaJ *	FE520065		1.62 (0.023)	1.58 (0.045)	1.60	0.034
*Down-regulated in metastasis*	FE519946		1.42 (0.004)	1.37 (0.010)	1.39	0.007
*Early-responsive to dehydration stress*	FE520632	1.54 (0.011)		1.37 (0.050)	1.45	0.031
*eIF3 subunit 5*	FE519503		1.36 (0.054)	1.34 (0.039)	1.35	0.047
*Embryo abundance protein*	FE518938		1.41 (0.043)	1.40 (0.031)	1.40	0.037
*Ethylene-forming enzyme*	FE519909		1.44 (0.020)	1.45 (0.022)	1.44	0.021
*FadR*	FE521144		1.50 (0.045)	1.62 (0.051)	1.56	0.048
*Ferredoxin *	FE519674		1.39 (0.054)	1.31 (0.002)	1.35	0.028
*FtsK*	FE520914		1.39 (0.006)	1.38 (0.039)	1.38	0.023
*Iodothyronine deiodinase 2*	FE519928		1.30 (0.054)	1.21 (0.051)	1.26	0.050
*Kinase-like protein*	FE521010		1.45 (0.050)	1.47 (0.054)	1.46	0.052
*LEA *	FE519754		1.37 (0.021)	1.44 (0.046)	1.40	0.034
*Methylenetetrahydrofolate reductase 1*	FE519965		1.61 (0.051)	1.40 (0.045)	1.51	0.043
*Mitochondrial import receptor *	FE519985		1.23 (0.045)	1.45 (0.052)	1.34	0.049
*Multidomain cyclophilin PPlases*	FE520902		1.34 (0.026)	1.31 (0.002)	1.33	0.014
*Pectate lyase*	FE520053		1.25 (0.035)	1.23 (0.052)	1.24	0.044
*Peroxidase*	FE522191	1.25 (0.012)		1.21 (0.036)	1.23	0.024
*Protein translation factor*	FE519467		1.28 (0.054)	1.24 (0.029)	1.26	0.042
*Pseudotzain*	FE520007		1.33 (0.040)	1.41 (0.052)	1.37	0.046
*Receptor-like protein kinase*	FE520561		1.22 (0.028)	1.28 (0.002)	1.25	0.015
*Sb50 fragment*	FE520247	1.25 (0.011)		1.30 (0.053)	1.28	0.032
*SINA fragment *	FE520708		1.33 (0.041)	1.43 (0.050)	1.38	0.046
*Subtilisin-like protease*	FE520006		1.27 (0.052)	1.33 (0.049)	1.30	0.051
*TCTP*	FE518564		1.21 (0.045)	1.33 (0.034)	1.27	0.045
*Translation factor SUI1*	FE519740		1.42 (0.049)	1.38 (0.029)	1.40	0.039

**^a^**Genbank accession number for the representing EST of each gene.**^b^**Three microarray experiments: Flynn-EW (earlywood in Flynn, spring), Flynn-LW (latewood in Flynn, autumn) and Kromelite-LW (latewood in Kromelite, summer). Gene expression ratios with (P-values) are presented.

## Discussion

### The mechanical properties of wood are complex traits

Tensile stiffness of xylem cells is largely determined by the angle of cellulose microfibrils in the middle layer (S2) of the secondary cell wall [14,15,43]. Wood stiffness has been shown to be under moderate to strong genetic control [35-39] and a number of QTLs for MOE or its major component traits (wood density and MFA) have been identified in a range of tree species [18-22,24]. The present study showed that wood stiffness had continuous variation with a nearly normal distribution at the family level, thus providing additional evidence that wood stiffness is a typical quantitative trait which is likely to be controlled by variation in numerous genes of small individual effect. Over 100 candidate genes were differentially transcribed in juvenile wood with contrasting stiffness and MFA, providing further molecular evidence that wood stiffness and MFA are complex traits controlled by numerous genes. The 3, 320 xylem unigenes on the microarrays were derived from relatively abundant xylem transcripts in radiata pine [42] and they may only represent 30-40% of genes moderately to highly transcribed during wood formation [44]. Therefore, the total number of genes influencing wood stiffness and MFA in radiata pine may be up to 2-3 times more than the number identified in this study.

The complexity of wood stiffness and MFA was also demonstrated by the relatively low magnitude of changes in transcript accumulation observed between juvenile wood with contrasting stiffness and MFA. In the three microarray experiments, the unigenes showing differential accumulation had average expression ratio values of 1.50 (Flynn-EW, spring), 1.46 (Flynn-LW, autumn) and 1.32 (Kromelite-LW, summer), respectively. These values are much lower than those observed in comparisons between EW and LW [45], and between juvenile and mature wood [46] using the same cDNA microarrays and experimental protocols. The smaller changes in transcript accumulation suggested that genes involved in the regulation of wood stiffness and microfibril orientation may individually have small effects.

### Cytoskeletal genes are implicated in stiffness and MFA

There is growing evidence that cortical microtubules, the dynamic arrays of alpha- and beta-tubulins in the cytoskeleton, play a key role during the crystallization of cellulose microfibrils [43] by directing their orientation during deposition in the wall [47]. In this study a *beta-tubulin *gene was preferentially transcribed in HS (low MFA) wood of radiata pine, providing molecular evidence that tubulins have functional roles in regulating MFA in the secondary xylem of gymnosperms. Several *alpha- *and *beta-tubulins *have previously been observed to be highly expressed in poplar xylem and tension wood with significantly reduced MFA [48]. Allelic variation in an *alpha-tubulin *was significantly associated with MFA in loblolly pine [27]. In *Eucalyptus nitens *a *beta-tubulin *gene was preferentially expressed in xylem from the upper sides of branches which had low MFA [49] and its over-expression in transgenic eucalypt xylem tissue directly influences MFA [25]. However, different beta-tubulin proteins have been observed to be expressed in both compression and opposite wood of maritime pine [50].

This study showed genes encoding actin and actin depolymerising factor (ADF) that were more strongly transcribed in juvenile wood with HS (low MFA) in radiata pine. ADF plays an important role in regulating the optimum balance between unpolymerised actin molecules and assembled actin filaments [51]. Actin filaments are one of the three major polymers in the cytoskeleton, but they are much less rigid compared to microtubules [10]. Actin microfilaments have been observed to align with cortical microtubules which in turn aligned with the orientation of cellulose microfibrils in cultured cotton fiber cells [52]. The *actin *and *ADF *genes identified in this study may affect wood stiffness and MFA by influencing interactions between actin filaments and microtubules.

### Secondary cell wall genes are implicated in stiffness and MFA

Candidate genes involved in secondary and primary cell wall formation may contribute to variation in MOE and MFA observed between trees. Many candidate genes preferentially transcribed in HS (low MFA) wood had clear roles in secondary cell wall formation. This included four secondary *cellulose synthases *(*PrCesA1, 3, 7, 11*) [42] and several genes involved in the biosynthesis of lignin (*C4H, C3H, CAD, PCBER*). In addition, transcripts (contigs) preferentially transcribed in HS (low MFA) juvenile wood in a single microarray experiment included many other secondary cell wall genes, such as *4CL, CCoAOMT, chitinase-like, SAMS, PPBG, AGP4, GRP2, PRP*, etc (data not show). In contrast, none of the candidate genes transcribed preferentially in LS wood (high MFA) have clear roles in secondary wall development. In fact, cell wall genes identified in juvenile wood with LS (higher MFA) were involved in cell division (*cyclin-like F-box*) and primary wall formation (*pectate lyase, peroxidase, cellulase *and *ovule/fiber cell elongation protein*). Taken together, increased secondary cell wall synthesis appears to be a fundamental process in the formation of HS (low MFA) wood.

Crystalline cellulose microfibrils are synthesised by cellulose synthase (CesA) complexes in the plasma membrane then extruded into the external matrix [53]. This study showed four secondary wall *PrCesA *genes and a *SuSy *gene that were transcribed more highly in HS wood with reduced MFA. Three other secondary *cellulose synthases *were present on the microarrays but were not differentially regulated (*PrCesA5, 6, 8*), suggesting that certain members of the *CesA *family may have specific roles associated with modification of cell wall stiffness and microfibril orientation. Previous studies showed that an over-expressed *SuSy *gene in poplar increased cellulose content, secondary wall thickness and wood density, but did not affect microfibril orientation [54]. However, a recent study identified an allelic variant in a radiata pine *SuSy *gene that was significantly associated with MFA [28].

Lignification is a distinct feature of secondary xylem development. Many lignin pathway or related genes were preferentially transcribed in HS (low MFA) wood, including *C4H, C3H, CAD *and *PCBER*, as well as several other genes that were identified in individual microarray experiments (*4CL, CCoAOMT*, and *SAMS*). More transcription of these genes in HS wood (with lower MFA) could increase lignification of secondary walls, resulting in higher density as seen in HS wood (Figure 2, 3 and Additional file 1). The lignified matrix in secondary walls can also generate compression resistance which additionally contributes to tensile stiffness of cells [43]. Antisense 4CL transgenic poplar produced low stiffness wood with reduced lignin content [55]. Associations of lignin genes with MFA have been observed in *Eucalyptus *(*CCR*) [30] and radiata pine (*PAL, COMT *and *PCBER*) [28], suggesting lignin genes may also be involved in the regulation of microfibril orientation. Previously, several genes involved in lignin biosynthesis have been found to be differentially regulated in the formation of tension wood [49,56] and compression wood [57] in which stiffness and MFA have been drastically altered. Taken together, candidate genes involved in lignin biosynthesis may influence both MFA and wood density through their key roles in secondary wall deposition.

### Hormone signalling genes associated with wood stiffness and MFA

It is well known that wood formation is largely regulated by different hormones. In the poplar xylem transcriptome approximately 2% of ESTs are involved in hormone biosynthesis [58]. Higher ethylene levels have been observed in compression and tension wood formation during which MFA is significantly altered [59], and an ACC oxidase protein involved in ethylene biosynthesis was more strongly expressed in compression wood [11]. Increased transcription of a gene encoding an ethylene-forming enzyme in high MFA wood and an *ethylene responsive element binding factor *(*ERF*) in low MFA wood was observed in this study. These data suggested that ethylene levels may affect MFA and wood stiffness via the transcription factor, *ERF*. The present study also identified a cytokinin-binding protein gene (*CBP*) that was more abundantly transcribed in HS wood (low MFA) in both summer and autumn. CBP has an essential role in cytokinin signal transduction pathways [60] and extra supply of cytokinin increases the formation of secondary xylem with higher lignification and thicker cell walls [61]. Thus, hormone signalling genes could be involved in the regulation of cellulose and lignin biosynthesis, and their differential expression may produce wood with contrasting stiffness and MFA. Identification of ethylene- and cytokinin-responsive genes in this study suggests their possible co-regulatory roles in juvenile wood formation leading to distinct stiffness and MFA. Interestingly, *phytochrome*, a light-responsive gene, was more abundantly transcribed in HS (low MFA) wood in radiata pine. *Phytochrome *regulates a large number of genes involved in hormone signalling or enzymes involved in cell wall modification [62]. However, the role of the *phytochrome *gene in the regulation of microfibril orientation and deposition remains unclear.

## Conclusions

Naturally occurring variation in microfibril orientation and mechanical properties of wood was correlated with distinct xylem transcriptomes, particularly in wood synthesized late in growing seasons (latewood). Genes involved in cytoskeleton development and secondary cell wall formation (cellulose synthesis and lignin pathway) were preferentially transcribed in secondary xylem with reduced microfibril angle and higher stiffness. In contrast, a few genes with a role in cell division and primary wall synthesis were more highly transcribed in wood with low stiffness and higher MFA. Many genes with unclear functions were also differentially regulated in wood with altered microfibril orientation and distinct mechanical properties. The identified candidate genes are a valuable resource for future transgenic studies and association analyses aimed at improving the mechanical properties of wood through manipulating cellulose microfibril orientation.

## Methods

### Field trials and selection of sampled trees

Two radiata pine (*Pinus radiata *D.Don) field trails planted at Flynn, Victoria (38° 14' S, 146° 45' E) and Kromelite, South Australia (37° 50' S, 140° 55' E) were used in this study. The two trials included 250 and 110 half- or full-sib families, respectively, and 16 full-sib families were common to both trials. At the time of sampling for wood property measurement and microarray experiments, the cambial ages (ring numbers at breast height) of the sampled trees in the Flynn and Kromelite trials were 7 and 6 yrs, respectively, both of which were producing juvenile wood. Tree diameters at breast height (DBH) were measured in both trials and overall wood stiffness of standing trees was assessed using an IML hammer (Instrument Mechanic Lab, Inc. Kennesaw, GA, USA) in the methods described previously [36]. The IML hammer provides a measurement of acoustic velocity (V) of an impulse to travel through the trunk of standing trees. Wood stiffness (MOE) was calculated based on the IML measurement: MOE = V^2 ^× 1000 [31].

Five families each with the highest and lowest MOE were selected at each site, on the basis of the IML-based measurement of standing trees. To maximize the difference between HS and LS trees in the comparisons, two individuals with higher (or lower) MOE were further selected in each of the five families with highest (or lowest) MOE. To account for the influence of growing conditions, all selected individuals had a straight bole with growth rates similar to or higher than the family average. The HS and LS trees being compared were also grown in a similar environment, such as no adjacent dead trees, similar soil and same slope direction. A total of 20 trees (5 families, 2 trees per family) in each trial were selected for microarray analysis.

### Measurement of microfibril angle and other wood traits

Wood cores sampled from 40 trees at the Flynn site and 60 trees at the Kromelite site were analysed at a high-resolution by SilviScan 2 [32,63]. These trees were selected from the HS and LS families identified above and included the trees used for microarray analysis. A wood core (12 mm in diameter) was drilled at breast height from each sampled tree. Cores were further trimmed to produce a 2 mm thick and 7 mm wide strip. MFA, wood density and MOE were measured across the wood strips. Wood trait variation based on SilviScan was compared between the two groups of sampled trees using average values in EW and LW of each ring, and P-values were calculated to indicate statistical significance.

### Collection of developing xylem tissues

Developing xylem tissues of selected trees were collected in the Flynn trial in spring (October) and autumn (April), when earlywood (EW) and latewood (LW) respectively, was being synthesized and from the Kromelite trial in summer (late November) when LW was being formed. The xylem tissues were scraped at breast height with a sharp chisel after removal of the bark. In the Flynn trial EW and LW tissues were collected on opposite sides of the trunk from the same trees. To avoid the presence of compression wood, developing xylem was collected from the tree trunk perpendicular to the prevailing wind direction. Fresh xylem tissues were immediately placed into liquid nitrogen in the field, and then stored at -80°C until RNA extraction.

### RNA isolation and microarray experiments

Total RNA was extracted using a modified CTAB method [64]. Radiata pine cDNA microarrays contained 6, 169 xylem ESTs assembled into 3, 320 unigenes (986 contigs and 2, 334 singletons) [42,45]. These unigenes were derived from genes moderately to highly transcribed in radiata pine wood formation [42] and may account for about 30-40% of the genes expressed in wood formation based on white spruce and poplar [44]. Probe synthesis, microarray hybridization and data normalization were performed in methods described previously [45]. Raw expression values from the 12 microarrays were deposited in the NCBI GEO database (accession number GSE23020).

Transcript accumulation was compared in juvenile wood with HS (low MFA) and LS (high MFA) using three microarray experiments: the Flynn trial in spring (EW) and in autumn (LW), and the Kromelite trial in summer (LW). In each experiment, total RNA samples extracted from the 10 HS wood were pooled into two bulks (five trees each, one tree per family) for comparisons with RNA pooled into two bulks of five LS wood. This pooling approach could partly account for the genetic variation among the different genotypes and provided two biological replicates for each experiment. A dye swap was performed for each biological replicate.

### Microarray data analysis and functional annotation

Wood stiffness is a quantitative trait [35-39] and is likely to be influenced by changes in expression of numerous genes. Some of these changes are expected to be small according to the multigenic model, in particular, the expression of transcription factors involved in the regulation of wood stiffness and microfibril orientation. Furthermore, gene expression usually changes in different biological replicates with various genetic backgrounds. Thus, a slightly lower threshold of transcript accumulation (20% change) and a moderately stringent average P-value (≤ 0.05) were used in this study to identify transcripts differentially accumulated in wood with distinct stiffness and MFA. Microarray expression of selected transcripts was validated using RT-MLPA (see below). Additional confidence in the identification of candidate genes was achieved by confirmation of the differentially accumulated transcripts in at least two of the three microarray experiments.

Identified differentially accumulated transcripts were functionally annotated using gene ontology (GO) terms [65]. Comparisons between transcripts differentially accumulated in wood with contrasting MFA and stiffness were performed according to the assigned GO term categories. GO terms differentially represented in HS (lower MFA) and LS (high MFA) wood (P-values ≤ 0.05) were identified using the "library comparison" function of the Bio301 system, an automated EST sequence management and functional annotation system [66].

### Validation of differentially accumulated unigenes

Microarray expression of differentially accumulated transcripts identified in the Flynn trial in autumn (Flynn-LW) were validated using the reverse transcriptase-multiplex ligation dependent probe amplification (RT-MLPA) method [67]. Eight genes preferentially transcribed in wood with HS and lower MFA, including *cellulose synthase 3 *(*PrCesA3*), *chitinase-like, protective protein for beta-galactosidase *(*PPBG*), *cinnamate 4-hydroxylase *(*C4H*), *phytochrome, PrCesA11, CCoAOMT *and *transmembrane serine protease 9 *(*TSP9*), and two genes preferentially transcribed in wood with LS and higher MFA (*FadR *and *T12C24.11*) were included in the validation (Additional file 3). Developing xylem tissues collected for the microarray experiment (Flynn-LW) were also used as the starting materials for the RT-MLPA validation. Eight biological replicates were used with four technical replicates. Differential transcription of each gene was summarized as a mean log-2 ratio from the 32 replicates.

Approximately 400 ng of DNase treated total RNA was reverse transcribed into first strand cDNA using the ImProm-II Reverse Transcription System (Promega, WI). The cDNA was hybridized at 60°C overnight with bulked RPO (right probe oligo) and LPO (left probe oligo) designed for the validated genes (Additional file 3). Ligation and PCR amplification were performed with SALSA D4 primer. Individual gene fragments were separated from the mixed PCR products using a CEQ™ 8000 Genetic Analysis System (Beckman Coulter, CA) and relative gene expression levels were determined using the built-in software.

## List of the abbreviations used

EW: earlywood; LW: latewood; HS: high stiffness; LS: low stiffness; MFA; microfibril angle; MOE: modulus of elasticity; QTL: quantitative trait loci; SNP: single nucleotide polymorphism; GO: gene ontology; RT-MLPA: reverse transcriptase multiplex ligation dependent probe amplification; DBH: diameter at breast height; CesA: cellulose synthase; SuSy: sucrose synthase; 4CL: 4-cinnamoyl CoA ligase; C3H: p-coumarate 3-hydroxylase; C4H: cinnamate 4-hydroxylase; CCR: cinnamoyl CoA reductase; CCoAOMT: caffeoyl CoA O-methyltransferase; COMT: caffeic acid O-methyltransferase; PAL: phenylalanine ammonia-lyase; PCBER: phenylcoumaran benzylic ether reductase; PPBG: protective protein for beta-galactosidase; SAMS: S-adenosylmethionine synthetase; AGP: arabinogalactan protein; FLA: fasciclin-like arabinogalactan protein; PRP: proline-rich protein.

## Authors' contributions

XL carried out cDNA microarray experiments, RT-MLPA validation, microarray and SilviScan data analyses, and manuscript preparation. HXW and SGS proposed the research project and guided the research process. All the authors have read and approved the manuscript.

## Supplementary Material

Additional file 1 **Variation of mechanical and other wood properties between the two groups of sampled trees in the Kromelite trial**. Microfibril angle (MFA), modulus of elasticity (MOE) and wood density in wood cores collected from the 20 sampled trees were measured by SilviScan 2. Wood variation between the two groups of sampled trees was compared in earlywood (EW) (a) and latewood (LW) (b) of each ring for MOE, MFA and density. Error bars represent the standard deviation of the mean value of each trait. Variation in MFA and MOE is statistically significant (P-values ≤ 0.05) but it is not significant for wood density variation except for ring 2 and 3 from the pith. Click here for file

Additional file 2 **A list of the 108 identified candidate genes**. Candidate genes were selected from differentially transcribed unigenes that were confirmed in at least two of the three microarray experiments. Click here for file

Additional file 3 **LPO (left probe oligo) and RPO (right probe oligo) of selected genes**. A total of 10 differentially transcribed genes identified in the microarray experiment using developing xylem collected in the Flynn trial in autumn were selected in the validation by reverse transcriptase-multiplex ligation dependent probe amplification (RT-MLPA). Click here for file
