# Supplementary figures and images for: Transcriptome profiling of Pinus radiata juvenile wood with contrasting stiffness identifies putative candidate genes involved in microfibril orientation and cell wall mechanics
Source: BMC Genomics. 2011 Oct 1;12:480. doi: 10.1186/1471-2164-12-480 (PMC3224210; doi:10.1186/1471-2164-12-480)

**(a)**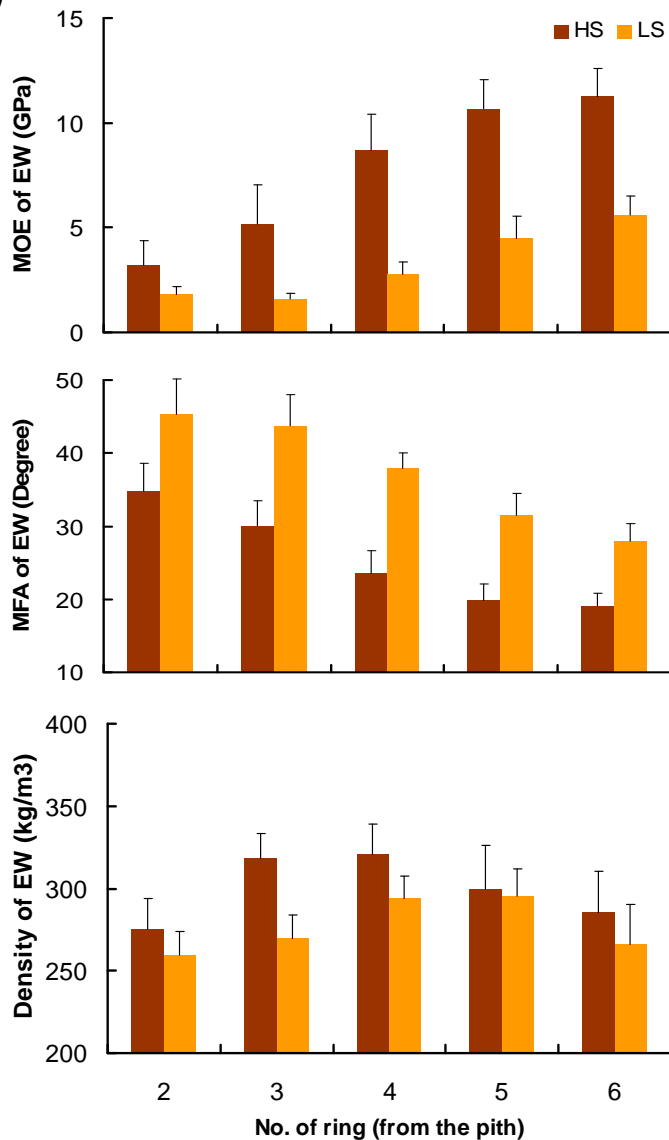**(b)**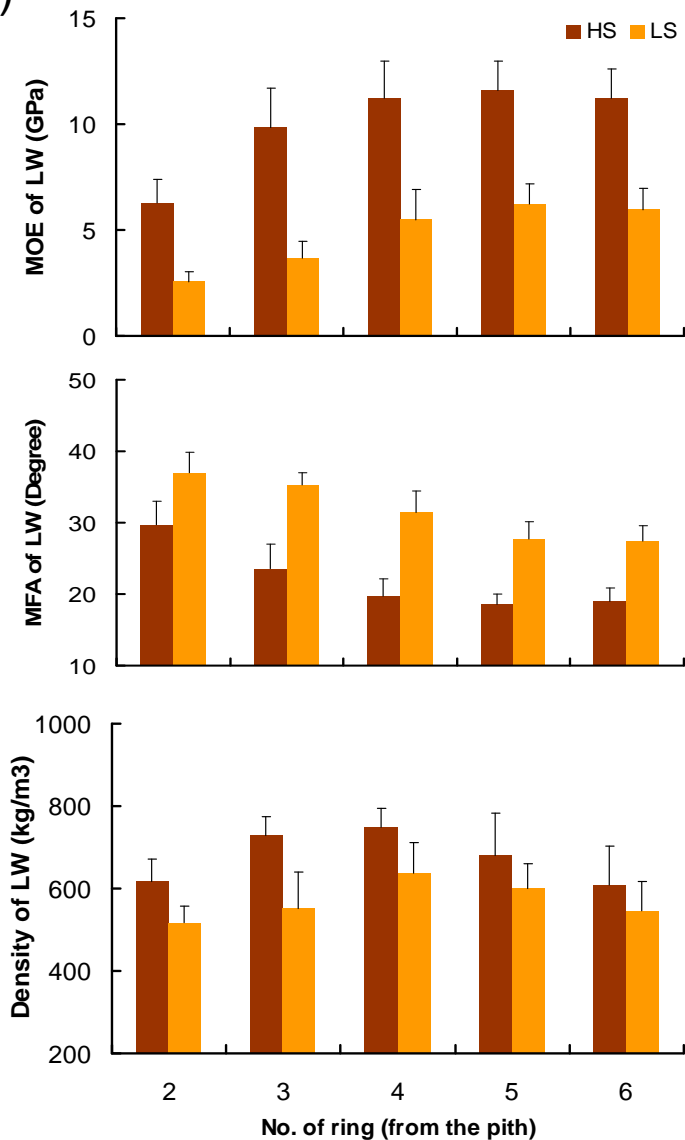

Supplement: Additional file 1 — Variation of mechanical and other wood properties between the two groups of sampled trees in the Kromelite trial. Microfibril angle (MFA), modulus of elasticity (MOE) and wood density in wood cores collected from the 20 sampled trees were measured by SilviScan 2. Wood variation between the two groups of sampled trees was compared in earlywood (EW) (a) and latewood (LW) (b) of each ring for MOE, MFA and density. Error bars represent the standard deviation of the mean value of each trait. Variation in MFA and MOE is statistically significant (P-values ≤ 0.05) but it is not significant for wood density variation except for ring 2 and 3 from the pith. [file 1471-2164-12-480-S1.PDF]
